# Defining a study population using enhanced reporting of Aboriginality and the effects on study outcomes

**DOI:** 10.23889/ijpds.v5i1.1114

**Published:** 2020-03-20

**Authors:** C McInerney, I Ibiebele, S Torvaldsen, JB Ford, JM Morris, M Nelson, D Randall

**Affiliations:** 1 The University of Sydney Northern Clinical School, Women and Babies Research, St Leonards, New South Wales, Australia; 2 NSW Biostatistics Training Program, NSW Ministry of Health, Australia; 3 Northern Sydney Local Health District, Kolling Institute, New South Wales, Australia; 4 School of Public Health and Community Medicine, UNSW Sydney, Australia; 5 Centre for Epidemiology and Evidence, NSW Ministry of Health, Australia

## Abstract

**Introduction:**

The under-reporting of Aboriginal and Torres Strait Islander people on routinely collected health datasets has important implications for understanding the health of this population. By pooling available information on individuals’ Aboriginal or Torres Strait Islander status from probabilistically linked datasets, methods have been developed to adjust for this under-reporting.

**Objectives:**

To explore different algorithms that enhance reporting of Aboriginal status in birth data to define a cohort of Aboriginal women, examine any differences between women recorded as Aboriginal and those assigned enhanced Aboriginal status, and assess the effects of using different reported populations to estimate within-group comparisons for Aboriginal people.

**Methods:**

Three algorithms, with different levels of inclusiveness, were used to establish different study populations all of which aimed to include all singleton babies born to Aboriginal or Torres Strait Islander women residing in New South Wales, Australia between 2010 and 2014 and their mothers. The demographics of the four study populations were described and compared using frequencies and percentages. In order to assess the impact on research outcomes and conclusions of using study populations derived from different algorithms, estimates of the associations between smoking during pregnancy and selected perinatal outcomes were compared using rates and relative risks.

**Results:**

Women included in the study population through enhanced reporting were older, less disadvantaged and more commonly resided in urban areas than those recorded as Aboriginal in the birth data. Although rates of smoking and some perinatal outcomes differed between the different study populations, the relative risks of each outcome comparing smoking and non-smoking Aboriginal mothers were very similar when estimated from each of the study populations.

**Conclusions:**

This work provides evidence that estimates of within-group relative risks are reliable regardless of the assumptions made for establishing the study population through the enhanced reporting of indigenous peoples.

## Background

Aboriginal and Torres Strait Islander Australians are under-reported on routinely collected health datasets nationwide [1], usually due to people not being asked in the standard way, or asked at all, whether they are Aboriginal and/or Torres Strait Islander, or choosing not to identify as such. Efforts are being made to improve data collection methods surrounding individuals’ Aboriginal and Torres Strait Islander status, including the release of national guidelines in 2010 for the collection of Indigenous status information specifically related to health data [2], and Council of Australian Governments agreement to implement the guideline improvements [3]. However, current under-recording has important implications for understanding Aboriginal and Torres Strait Islander health and consequently for the evaluation of targeted policy and delivery of health services [1]. New South Wales (NSW) data collections are no exception and the estimated degree of under-recording varies substantially for each dataset [4]. The degree of the problem is difficult to determine, but a survey validation study found that 90.7% of Aboriginal and Torres Strait Islander people were correctly recorded on NSW hospital records in 2010 [5], and a comparison between recording on NSW perinatal data and an enhancement algorithm estimated that 71.3% of Aboriginal mothers were recorded as such on the perinatal data in 2010 [6].

For the purposes of this study, Aboriginal and Torres Strait Islander people were grouped together because of the small proportion of Torres Strait Islander people living in NSW (an estimated 2.6% of all females of Aboriginal and/or Torres Strait Islander descent [7]) and some people are recorded as both. We respectfully use the term Aboriginal as Aboriginal people are the original inhabitants of NSW [8].

With the increasing availability of timely linked datasets, many research groups enhance reporting by pooling available information on an individual’s Aboriginality from linked datasets to assign an enhanced Aboriginal status. This technique has been coined the Enhanced Reporting of Aboriginality (ERA) [9]. Many algorithms have been developed for the ERA, each requiring varying degrees of evidence to assign an individual enhanced Aboriginal status. Algorithms that require verification of a person’s Aboriginality from a number of independent sources are more likely to correctly report a person as Aboriginal but also more likely to incorrectly assume someone is not Aboriginal; conversely more inclusive algorithms are increasingly susceptible to data entry or linkage errors [1]. Estimating the burden of adverse health outcomes among Aboriginal people is therefore dependent on the algorithm used and a number of studies have shown this can also affect estimates of prevalence and incidence [10]. This has major implications for the National priority on Closing the Gap on Indigenous Health Outcomes [11] as measuring the gap is not straight-forward.

Although it is important to understand the health disadvantage experienced by Aboriginal people, there is growing opinion that comparisons to Non-Aboriginal Australians are not always helpful and comparisons among Aboriginal people may be more constructive [12, 13]. Currently little is known about how different methods of ERA affect these within-group comparisons. The aims of this study were to: (1) establish a cohort of women who were recorded as Aboriginal on the baseline dataset, in this case the NSW birth data (all of whom were assumed to be Aboriginal); (2) explore a range of algorithms to enhance Aboriginal status using additional datasets; and (3) examine any differences in research outcomes when using different reported populations to estimate within-group comparisons for Aboriginal people, specifically the associations between smoking during pregnancy and perinatal outcomes. 

## Methods

Three algorithms were used to establish different study populations, all of which aimed to include all singleton babies born to Aboriginal or Torres Strait Islander women residing in NSW between 1 January 2010 and 31 December 2014 and their mothers. This date range was chosen in order to have a full five years of data for all datasets in the study, to ensure adequate population size and also have the most up-to-date data available at the time. Each algorithm assessed the evidence available in each dataset and, based on predefined constraints, assigned each mother an enhanced Aboriginal status.

### Data Sources

All datasets used in this study are held by the NSW Government and routinely collected for administrative and/or surveillance purposes. The study population was identified from all records in the NSW Perinatal Data Collection (‘birth data') for the period 1 January 2010 to 31 December 2014. This surveillance system (previously called the Midwives Data Collection, and established more than 30 years ago) includes all live births and stillbirths of at least 400 grams birthweight or at least 20 weeks gestation, including births at NSW public and private hospitals and home births [14]. Additional birth data, including all records from 1 January 2001 to 31 December 2014, were used for the enhanced reporting, to ascertain records for previous births to the mothers identified in the study population. Information surrounding the mother’s Aboriginality is collected independently for each birth by the midwife and recorded in the birth data [4].

Public and private hospital admission records were drawn from the Admitted Patient Data Collection (‘hospital data’; previously the Inpatient Statistics Collection, and established more than 35 years ago) for admission dates from 1 January 2001 to 31 December 2015. Diagnoses coded in the hospital data are applied according to the International Classification of Diseases, Australian Modification (ICD-10-AM). Information on all visits to a public emergency department was taken from the NSW Emergency Department Data Collection (‘emergency data’) between 1 January 2005 and 31 December 2015. The collection of emergency data in NSW commenced in 1994, and became an organised collection from July 1996, with the number of participating emergency departments increasing each year to around 90 in 2010, covering the majority of the NSW population [15]. Data on a person’s Aboriginality is collected independently for each hospital and/or emergency department visit [4]. Under the Births, Deaths and Marriages Registration Act 1995, parents must register their child’s birth with the Registry of Births, Deaths and Marriages (‘birth registration data’) in order for a birth certificate to be produced [15]. All births within NSW should be registered with basic demographic information including parents’ Aboriginal status by the family. Birth registration data between 1 January 2001 and 31 December 2015 were used for the enhanced reporting. For all datasets in this study, the national standard question and recording categories are used to collect information surrounding an individual’s Aboriginal status [16].

Records within and across all datasets were probabilistically linked using personal identifiers such as name, address and date of birth, by the NSW Centre for Health Record Linkage (CHeReL). The CHeReL uses the Choicemaker software for linkage, which uses blocking and machine learning to assign weights to potential matches [17], and has an estimated false linkage rate of less than 5 per 1,000 records [18]. Hospital birth records were those where the date of birth from the linked birth data was between the mother’s admission and discharge dates. Where transfers or changes of care occurred during the birth admission, multiple records were identified as hospital birth records. In this case, these records were collapsed into one hospital stay for use in the ERA algorithms, as Aboriginal status is generally recorded once per hospital stay [4]. 

### Algorithms

All women with a birth record in the NSW birth data in the study period were potentially eligible for inclusion in the study, either in the baseline population or in one of the enhanced populations. Due to the previously noted under-reporting of Aboriginal mothers in the birth data, a number of ERA algorithms were applied to increase ascertainment of eligible participants, resulting in different reported populations. Since we wanted to *enhance* the reporting of Aboriginality among those assessed for eligibility, all women recorded as Aboriginal on the birth data were assumed eligible for inclusion in all study populations regardless of their Aboriginal status on other linked records. Thus the following algorithms were only applied to those women who were not recorded as Aboriginal on the current birth data.

Study population 1 was established using the least inclusive algorithm and inclusiveness increased with population number. 

#### Study population 1

Study population 1 consisted of just those mothers who were recorded as Aboriginal on the birth data and their babies, thus providing a lower bound on all study populations.

#### Study population 2

**Figure 1: A flow diagram demonstrating the logic used to enhance the reporting of Aboriginality and define study population 2:**
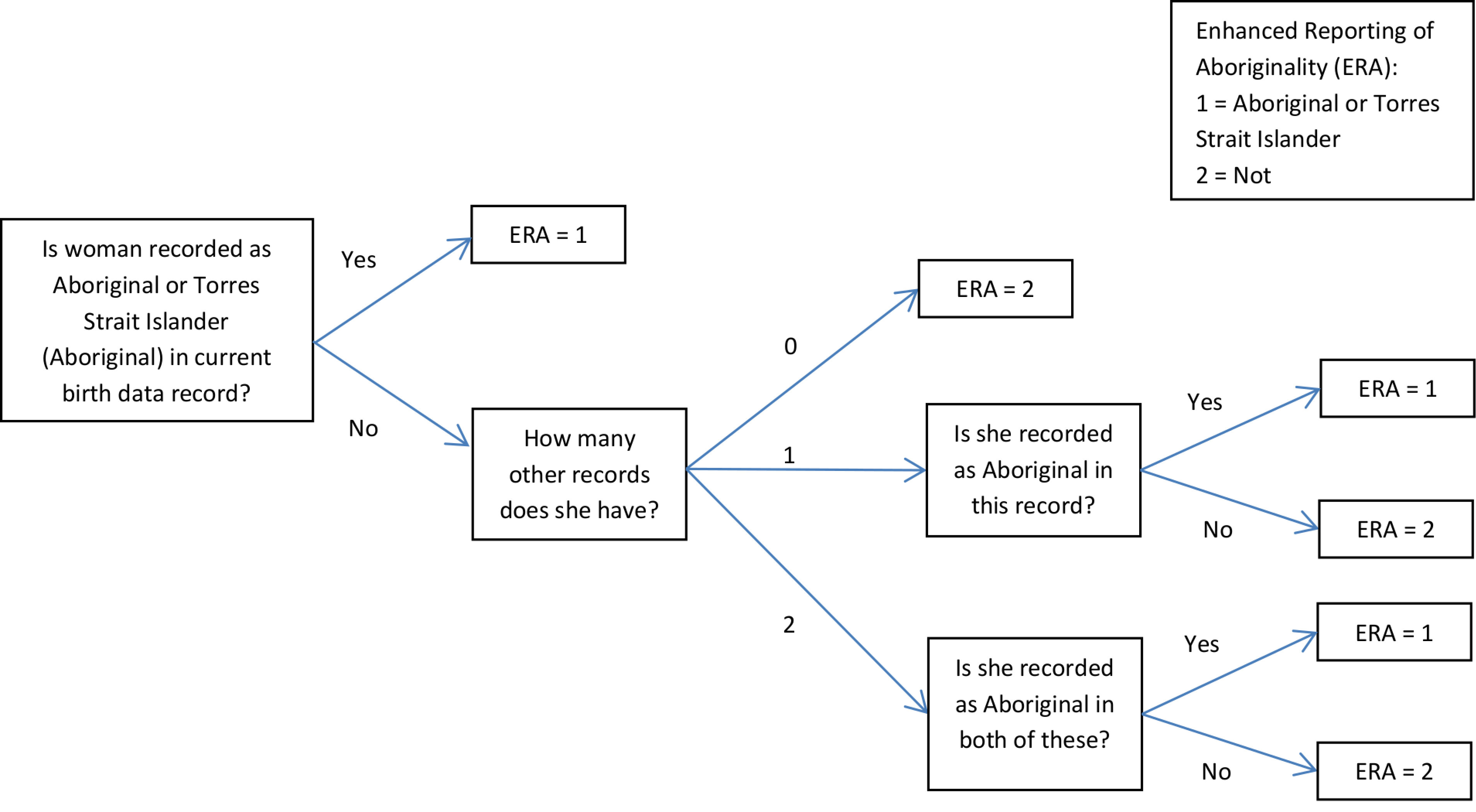


Study population 2 was established using only the data surrounding the birth; this included the birth data and, where available, the corresponding hospital birth record and birth registration. The recording of any woman without a corresponding hospital birth record or registration (and not recorded as Aboriginal in the birth data) could not be further enhanced and was thus not included in study population 2. Where the hospital birth record and birth registration were available, both needed to indicate that the mother was Aboriginal for enhanced inclusion in study population 2. If the mother only had one linked record (either the hospital birth record or the birth registration), that record needed to indicate she was Aboriginal for enhanced inclusion in the study population. A flow diagram outlining the logic of this algorithm is given in Figure 1.

In the case where multiple records were identified as hospital birth records and where Aboriginality differed on these records, a relatively conservative method was used to collapse these into one hospital stay for use in the algorithm:

- if the mother was recorded as Aboriginal *and* non-Aboriginal on at least one hospital birth record respectively, then she was excluded from the enhanced population;
- if at least one hospital birth record for the mother was recorded as Aboriginal and all remaining hospital birth records were missing or Aboriginal status was recorded as unknown, these records were collapsed into one that reported the mother as Aboriginal.

Babies born from a multiple birth who were assigned Aboriginal status from the ERA but their twin was not (‘lonely twins’) were excluded from the enhanced population.

#### Study population 3

To establish study population 3 a Multistage-Median algorithm[19] was applied to all linked records from the birth, hospital (all records, not just the birth record), emergency and birth registration data. The Multistage-Median method initially assigns a dataset-specific Aboriginal status to all mothers using multiple records within the one dataset. A similar algorithm is then used to assign an overall status using the multiple statuses from each dataset the woman appears in. In more detail, a woman was assigned Aboriginal status in a dataset if:

- she had one or two linked records and at least one recorded her as Aboriginal,
- she had three or more linked records and at least two recorded her as Aboriginal

**Figure 2: A flow diagram demonstrating the logic used to enhance the reporting of Aboriginality and define study population 3.**
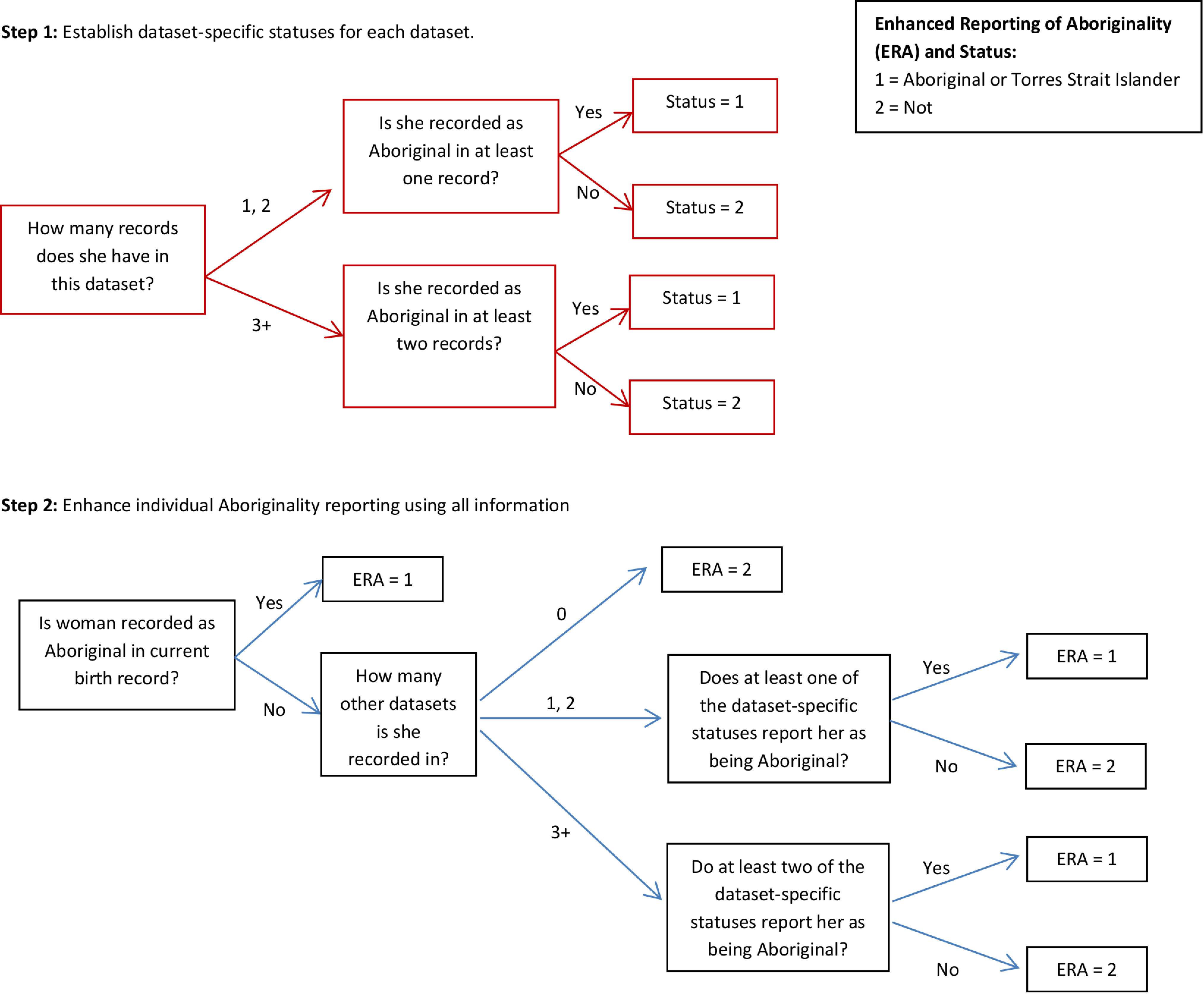


A comparable algorithm using dataset-specific statuses instead of records was used to assign an overall status and thus determine the inclusion of each woman in study population 3. A flow diagram outlining the logic of this algorithm is given in Figure 2.

Where a mother had multiple records for one stay in the hospital data and where Aboriginality differed on these records, these records were collapsed into one hospital stay for use in the algorithm. An algorithm with the same logic used to establish each data-set specific status was used to assign an individual status for one hospital stay where a mother had multiple hospital birth records from one birth.

#### Study population 4

Study population 4 included all mothers that were recorded as Aboriginal on at least one record from the birth, hospital, emergency or birth registration data. For the hospital data, this included all linked records, not just the linked birth record.

### Study exposure, outcomes and covariates

The study exposure was maternal smoking status. The birth record and the mother’s hospital birth data were used to assign smoking status. If the birth data indicated that a mother smoked at any time during her pregnancy and/or if she was recorded as a current smoker on her hospital birth record(s) (ICD-10-AM diagnosis codes Z72.0 and F17) then she was considered to be a smoker. Smoking is self-reported in both datasets and is known to be under-reported with an estimated sensitivity and specificity of 58.5% and 98.4% respectively in the hospital data [20].

The outcomes of interest included perinatal death (stillbirth and neonatal death), preterm birth (<37 completed weeks of gestation), and small-for-gestational age (birthweight <10th percentile for sex and age [21]).

Study covariates included maternal age, any hypertension, any diabetes, parity and socio-economic status. Maternal age and parity were reported according to the birth data. The mother’s chronic conditions [22], hypertension and diabetes information were obtained from the birth and hospital birth record(s). The NSW ranking of the Australian Bureau of Statistics (ABS) 2011 Socio-Economic Index for Areas (SEIFA) Index of Relative Socio-Economic Disadvantage (IRSD) and the 2011 Remoteness Areas were used to assess the mother’s relative socio-economic status and access to services respectively. Where available, the mother’s 2011 Statistical Local Area (SLA) according to her birth data was used to assign these measures. Otherwise, and for all babies born in 2010, the mother’s 2010 SLA was used. Hospital type, an indicator of both hospital size and location (urban or regional) [23], was assigned using the hospital code recorded in the birth data.

### Statistical analysis

The demographics of each of the four study populations and separately for those included via the ERA algorithms were described and compared using frequencies and percentages. The additional women included through the ERA in study population 2, 3 and 4 are referred to as ERA 2, 3 and 4 respectively.

In order to assess the effects on health outcomes and comparisons of using study populations derived from different algorithms, estimates of the associations between smoking during pregnancy and selected perinatal outcomes were compared using rates and relative risks. To estimate the unadjusted and adjusted relative risk of each binary outcome while accounting for the correlation within the data (some mothers had more than one baby during the study period), an extension to the modified Poisson regression [24] was used with an unstructured correlation matrix. SAS version 9.4 (SAS Institute, Cary, NC, USA) was used for all data manipulation and analysis. 

## Results

Following exclusion of duplicates (n=76), a total of 487,388 babies were born to 379,116 mothers in NSW and were assessed for inclusion in this study. Aboriginal status was not stated for 623 mothers (0.13% of all births). All 470,484 babies born to mothers not recorded as Aboriginal in the birth data (either Non-Aboriginal or not stated) were assessed for inclusion using each of the ERA algorithms.

### Study population 1

Birth data records indicated that 16,904 babies were born to 12,720 Aboriginal mothers between 2010 and 2014 in NSW. After excluding all babies from multiple births, those born to mothers who were interstate residents at the time of birth or those not recorded as Aboriginal, study population 1 consisted of 16,306 singleton babies born to 12,504 Aboriginal mothers (Figure 3).

**Figure 3: Flow diagram of mothers and babies eligible for inclusion in study population 1. Aboriginality of mothers in this study population was based only on what is recorded in the birth data; no enhanced reporting of Aboriginality was used.**
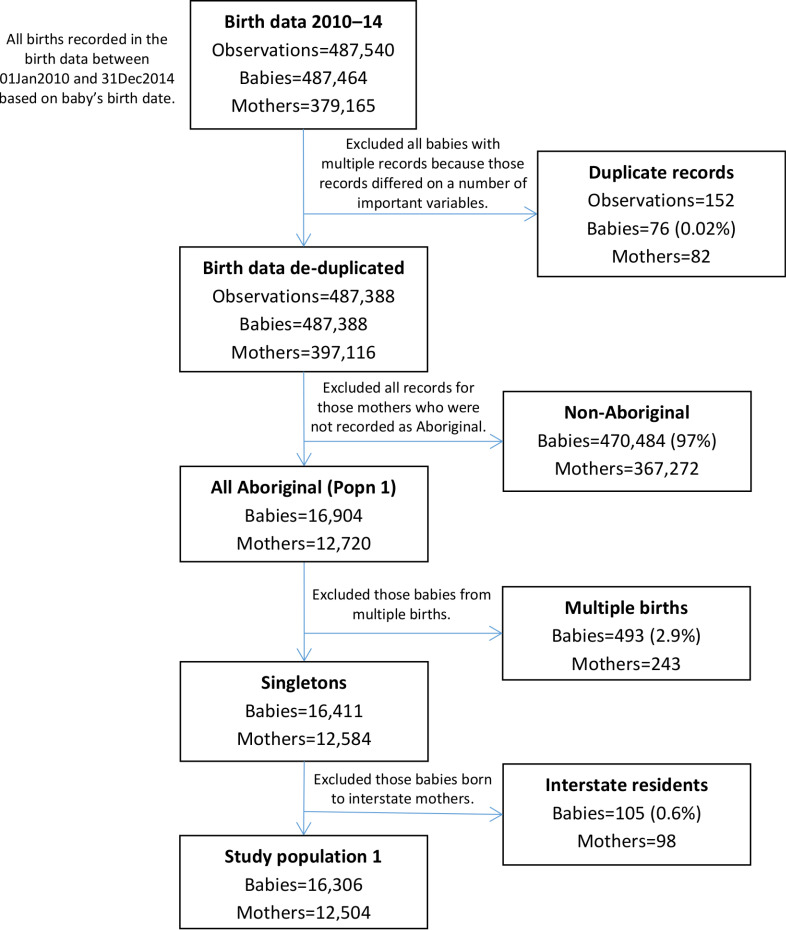


### Study population 2

885 babies born to 779 mothers were assigned enhanced Aboriginal status using this algorithm; making a total population of 17,789 babies born to 13,267 Aboriginal mothers (Figure 4). Almost all births in the birth data linked to a hospital birth record (99%) and the vast majority linked to a birth registration (97%). After exclusion of all babies from multiple births and those born to interstate residents, study population 2 consisted of 17,175 babies (5% from the ERA) born to 13,049 mothers.

**Figure 4: Flow diagram of mothers and babies eligible for enhanced inclusion in study population 2. Aboriginality of mothers in this study population was based on that recorded in the birth data and the enhanced reporting of Aboriginality from linked data records related to the birth.**
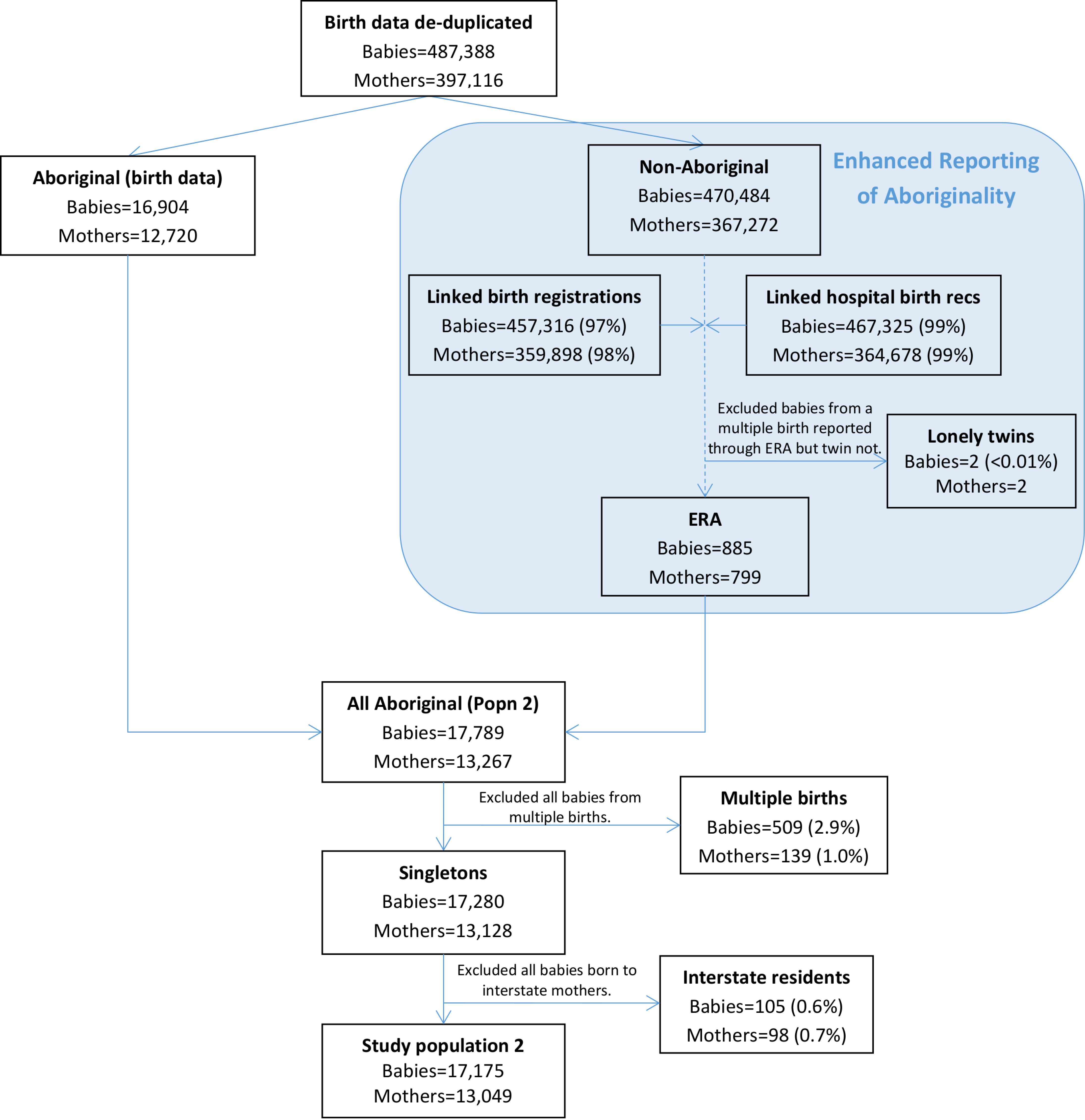


### Study population 3

Almost all mothers not recorded as Aboriginal on the birth data were assigned dataset-specific statuses regarding Aboriginality for the hospital (99.7%) and birth registration data (99%) and 67% were assigned a dataset-specific status for the emergency data (Figure 5). 1,921 babies born to 779 mothers were assigned enhanced Aboriginal status using this algorithm and these babies made up 10% of final study population 3. After exclusion of all babies from multiple births and those born to interstate residents, study population 3 consisted of 18,154 babies born to 13,477 mothers.

**Figure 5: Flow diagram of mothers and babies eligible for inclusion in study population 3. Aboriginality of mothers in this study population was based on that recorded in the birth data and the enhanced reporting of Aboriginality using the multi-stage median algorithm and all available linked data.**
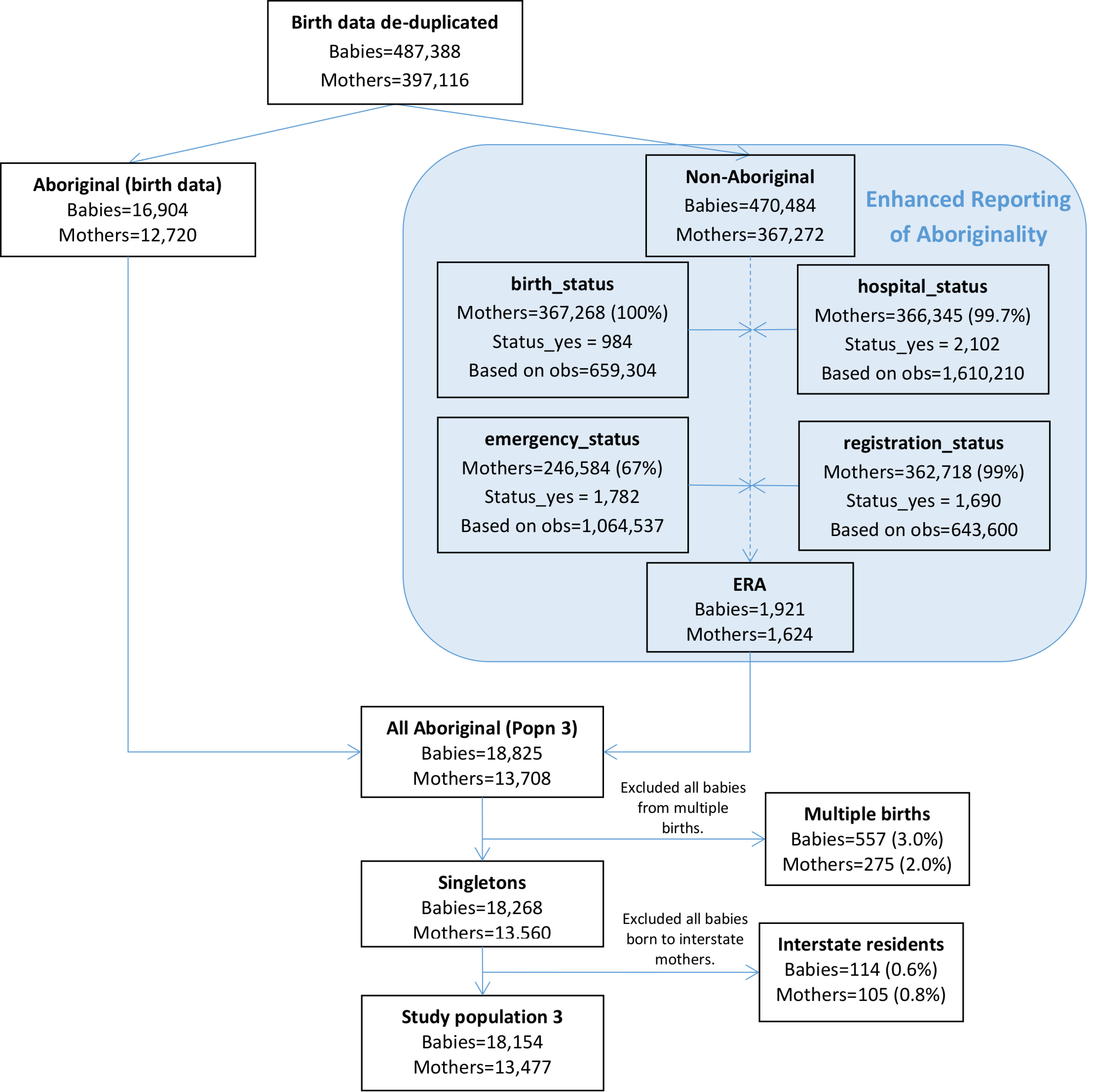


### Study population 4

Almost all mothers not recorded as Aboriginal on the birth data had at least one record in the hospital (99.7%) and birth registration data (99%) and 67% had at least one record in the emergency data (Figure 6). 8,143 babies born to 6,442 mothers were assigned enhanced Aboriginal status using this algorithm and these babies made up 33% of the final study population 3. After exclusion of all babies from multiple births and those born to interstate residents, study population 3 consisted of 24,107 babies born to 17,947 mothers.

**Figure 6: Flow diagram of mothers and babies eligible for inclusion in study population 4. Aboriginality of mothers in this study population was based on ever being recorded as Aboriginal in any of the available linked data.**
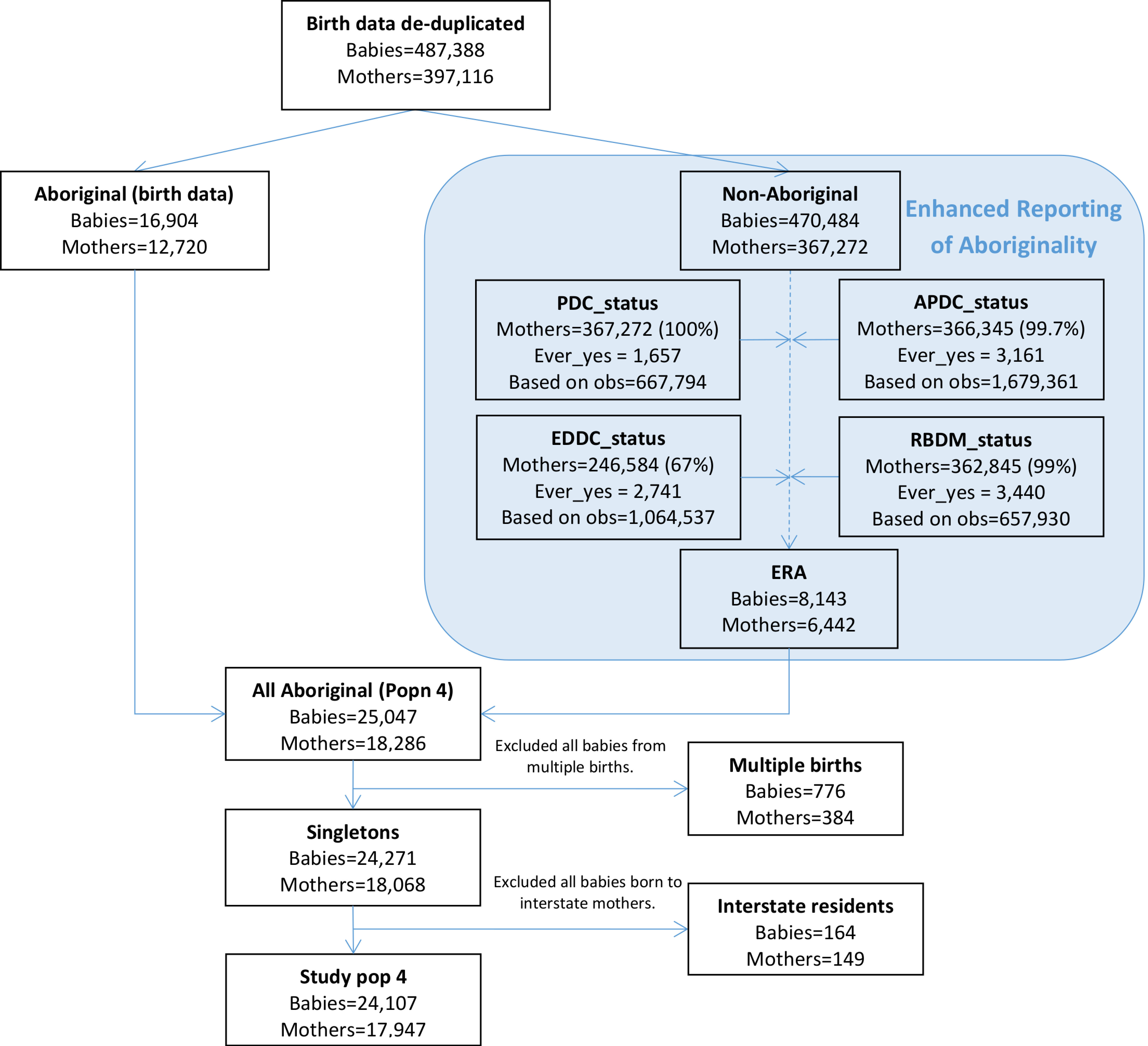


### Comparison to Mothers and Babies report

The number of births to mothers recorded as Aboriginal on the birth data increased from 2011 (N=2,975) to 2014 (N=3,754). The increase in births when applying the ERA was not consistent over the study period; falling from a 6% to 3% increase for population 2, a 16% to 6% increase for population 3 and a 58% to 36% increase for population 4 (Table 1). Similarly, the estimated level of reporting of the Aboriginality of mothers on the birth data increased over the study period (Appendix 1).

**Table 1: Number (N) of births to Aboriginal mothers in 2010 to 2014 (including multiple births and births to interstate residents) according to the four study populations and the percent increase from study population 1.** 

	Year									
	2010		2011		2012		2013		2014	
	N		N		N		N		N	

Population 1	3,088		2,975		3,348		3,492		3,754	
	N	Incr. %	N	Incr. %	N	Incr. %	N	Incr. %	N	Incr. %
Population 2	3,277	6	3,188	7	3,541	6	3,659	5	3,856	3
Population 3	3,570	16	3,459	16	3,758	12	3,794	9	3,965	6
Population 4	4,871	58	4,720	59	5,000	49	4,963	42	5,104	36

### Demographics

Women reported through the ERA tended to be older, less disadvantaged and more commonly resided in urban areas than study population 1 (Figure 7). A maternal age of 20-24 years was most common among mothers from study population 1 (34%), ERA2 (30%) and ERA3 (30%), while ERA4 tended to be older with 28% of mothers having a maternal age of 25-29 years. Over one quarter of women from study population 1 were in the most disadvantaged quintile (27%), compared to 22-24% of women recorded through the ERA. Over a third of women from the ERA 4 group resided in major cities (37%) and considerably more women from the ERA 2 and 3 groups resided in major cities (28% and 30% respectively) than those from study population 1 (22%). The proportion of women that smoked during pregnancy was lower among mothers recorded through the ERA groups than those from study population 1 (Table 2). Hypertension and chronic conditions were similar among all mothers however the prevalence of diabetes was lower among women from the ERA groups than those from study population 1.

**Table 2: Smoking and health status at the time of birth of mothers who were recorded as Aboriginal in the birth data (Population 1) and from an enhanced reporting of Aboriginality algorithm (ERA2 – 4) who gave birth to at least one singleton baby in NSW between 2010 and 2014.** ^Chronic conditions encompasses renal, cardiac, thyroid, asthma, psychiatric, and other autoimmune conditions[22].

	Population 1	ERA 2	ERA 3	ERA 4
	N=16,306	N=869	N=1,848	N=7,801
	%			
		
Smoked during pregnancy	49.7	41.2	43.8	32.5
Chronic conditions^	1.8	1.2	2.5	1.7
Any hypertension	9.4	8.8	8.6	9.2
Any diabetes	7.9	5.6	6.4	7.2

**Table 3: Demographics at the time of birth of all Aboriginal or Torres Strait Islander mothers who gave birth to at least one singleton baby in NSW between 2010 and 2014 by method of reporting of Aboriginality.** *Socio-Economic Index for Areas – Index of Relative Socio-Economic Disadvantage (SEIFA IRSD). When ranking areas within NSW in order of their relative disadvantage, the lowest 20% (most disadvantaged) fall in the 1^st^ quintile and the highest 20% (least disadvantaged) fall in 5^th^ quintile. ^Chronic conditions encompasses renal, cardiac, thyroid, asthma, psychiatric, and other autoimmune conditions[22].

	Population 1	Population 2	Population 3	Population 4
	N=16,306	N=17,175	N=18,154	N=24,107

Maternal age	%	%	%	%

Under 20	18.0	18.1	17.7	15.6
20–24	33.5	33.3	33.1	30.6
25–29	25.1	25.2	25.4	25.9
30–34	14.8	14.8	15.0	17.0
35 and over	8.7	8.7	8.8	10.9
Parity	N=16,300	N=17,169	N=18,147	N=24,093

0	34.8	35.2	34.5	34.3
1	25.5	25.4	26.0	26.2
2	17.3	17.1	17.1	17.7
3+	22.4	22.2	22.4	21.8
SEIFA IRSD quintiles*	N=16,223	N=17,089	N=18,059	N=23,988

1st – most disadvantaged	27.1	26.8	26.7	25.7
2nd	20.5	20.4	20.3	19.7
3rd	29.8	29.8	29.8	28.5
4th	16.7	17.0	17.0	17.7
5th – least disadvantaged	5.8	6.0	6.2	8.4
Remoteness area	N=16,222	N=17,088	N=18,059	N=23,989

Major cities	22.5	22.8	23.2	27.2
Inner regional	33.9	33.8	34.0	34.0
Outer regional	34.0	34.1	33.8	31.3
Remote	6.1	5.9	5.7	4.9
Very remote	3.5	3.4	3.3	2.7
Smoked during pregnancy	N=16,306	N=17,175	N=18,154	N=24,107
Yes	49.7	49.3	49.1	44.2
Chronic conditions^	N=16,306	N=17,175	N=18,154	N=24,107
Yes	1.8	1.8	1.9	1.8
Any hypertension	N=16,306	N=17,175	N=18,154	N=24,107
Yes	9.4	9.3	9.3	9.3
Any diabetes	N=16,306	N=17,175	N=18,154	N=24,107
Yes	7.9	7.8	7.8	7.7

**Figure 7: Demographics at the time of birth of mothers who were reported as Aboriginal on the current birth data (Pop 1) or from an enhanced reporting of Aboriginality algorithm (ERA1 – 3) who gave birth to at least one singleton baby in NSW between 2010 and 2014.**
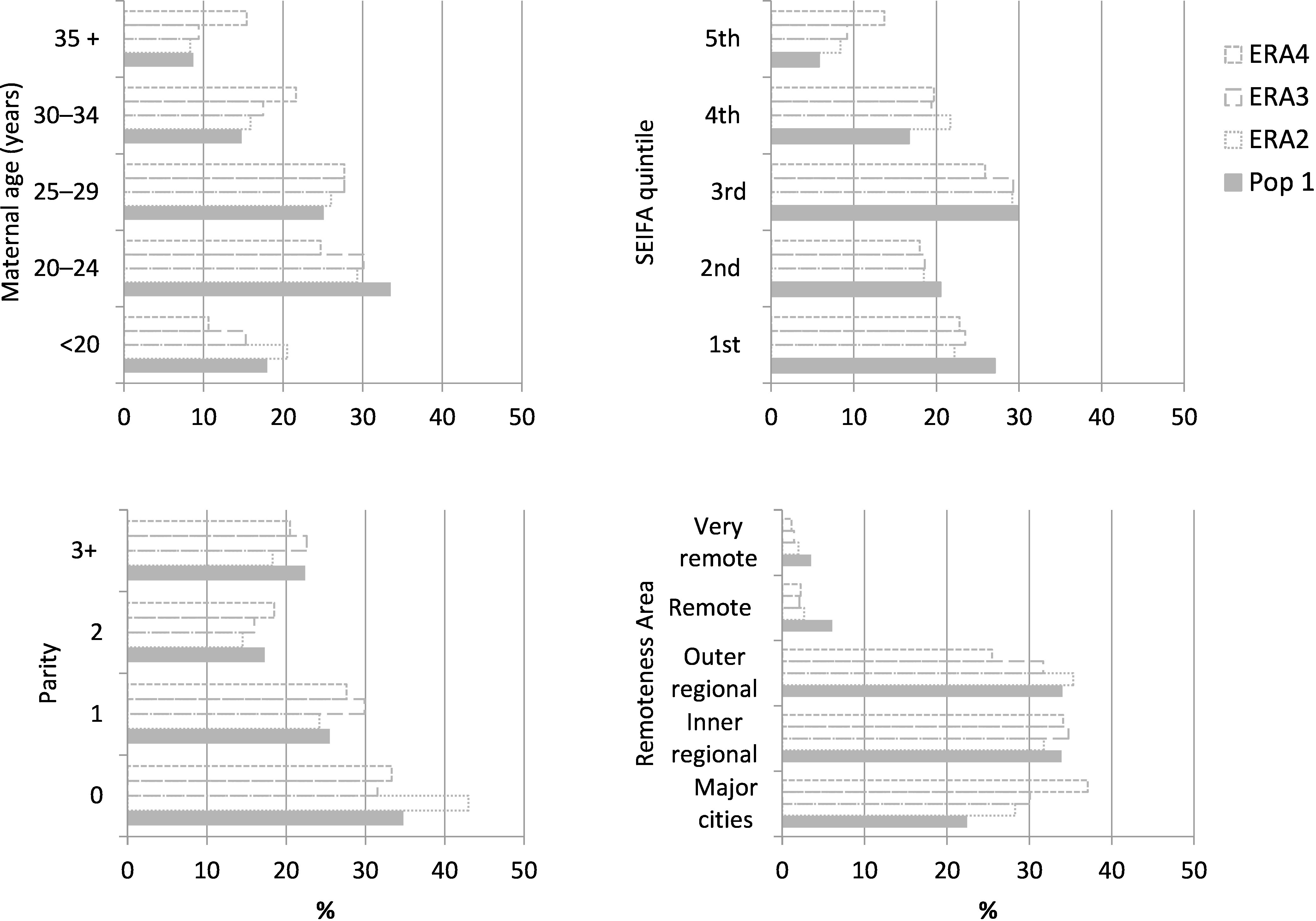


### Perinatal outcomes

Regardless of the study population, adverse perinatal outcomes occurred less frequently among babies born to non-smoking mothers (Table 4). Each estimate of prevalence of preterm birth, small-for-gestational age (SGA) and perinatal death among babies born to smoking and non-smoking mothers was within 1% of that from all study populations. The unadjusted and adjusted relative risks of each outcome were almost exactly the same when estimated from each of the 4 study populations.

**Table 4: Frequency of perinatal outcomes at the time of birth of all Aboriginal mothers by smoking status during pregnancy and the estimated relative risk (RR) with 95% confidence intervals (95% CI).** *adjusted for maternal age, any hypertension, any diabetes, parity and socio-economic status (SEIFA).

Study population	N	%	RR (95% CI)	
			Unadjusted	Adjusted*

Preterm birth				

1	16,306	11.2		
Smokers	8,109	14.2	0.58	0.58
Non-smokers	8,197	8.1	(0.53, 0.64)	(0.53, 0.64)
2	17,175	11.1		
Smokers	8,467	14.2	0.58	0.59
Non-smokers	8,708	8.1	(0.53, 0.64)	(0.53, 0.64)
3	18,154	11.3		
Smokers	8,919	14.4	0.59	0.58
Non-smokers	9,235	8.2	(0.54, 0.64)	(0.53, 0.64)
4	24,107	10.3		
Smokers	10,646	13.6	0.58	0.58
Non-smokers	13,461	7.7	(0.54, 0.63)	(0.54, 0.63)
Small-for-gestational age (<10th percentile)				

1	16,306	13.3		
Smokers	8,109	19.7	0.36	0.35
Non-smokers	8,197	7.0	(0.33, 0.39)	(0.32, 0.38)
2	17,175	13.2		
Smokers	8,467	19.6	0.36	0.35
Non-smokers	8,708	7.0	(0.33, 0.39)	(0.32, 0.38)
3	18,154	13.1		
Smokers	8,919	19.5	0.36	0.35
Non-smokers	9,235	7.0	(0.33, 0.39)	(0.32, 0.39)
4	24,107	12.5		
Smokers	10,646	19.3	0.37	0.36
Non-smokers	13,461	7.1	(0.35, 0.40)	(0.33, 0.39)
Perinatal death				

1	16,306	1.4		
Smokers	8,109	1.8	0.53	0.57
Non-smokers	8,197	1.0	(0.40, 0.70)	(0.42, 0.75)
2	17,175	1.4		
Smokers	8,467	1.8	0.55	0.58
Non-smokers	8,708	1.0	(0.42, 0.71)	(0.44, 0.77)
3	18,154	1.4		
Smokers	8,919	1.8	0.54	0.58
Non-smokers	9,235	1.0	(0.42, 0.70)	(0.44, 0.76)
4	24,107	1.3		
Smokers	10,646	1.7	0.54	0.57
Non-smokers	13,461	0.9	(0.43, 0.68)	(0.45, 0.73)

## Discussion

We applied a number of algorithms to enhance the reporting of Aboriginality in the NSW birth data, thus increasing the size of the study population for subsequent analysis. As expected, the size of the study population increased with the inclusiveness of the algorithm used. The population size ranged from 16,306-24,107 babies born to 12,504-17,947 Aboriginal mothers respectively. Interestingly, the increase in women reported as Aboriginal through the ERA dropped over the study period for all algorithms and conversely the level of reporting increased. The percent increase in births observed when applying the different ERA algorithms also decreased over time. This could be due to measures that have been taken to improve recording in hospitals and observed increases in the propensity to identify as Aboriginal, meaning there is less potential for the ERA to add more information.

The demographics of women assigned enhanced Aboriginality from the three ERA algorithms differed from each other and those recorded as Aboriginal on the birth data. Generally, women recorded through the ERA were older, less disadvantaged, lived in less remote areas and were less likely to smoke or have diabetes than women recorded as Aboriginal on the birth record. Overall, these differences increased with the inclusiveness of the ERA algorithm. These findings are consistent with a similar NSW study which found that ERA tended to capture more women from urban areas than rural areas (but did not report on other demographics) and a study from Western Australia which concluded that Aboriginal or Torres Strait Islander babies reported through the ERA were less disadvantaged [4, 25]. The age differences are likely related to the increase in urban and less disadvantaged women in the enhanced populations, as women of higher socio-economic status who give birth in urban areas tend to be older than women in rural and more disadvantaged areas.

It is important that every individual has a choice whether or not to identify themselves as Aboriginal or Torres Strait Islander, and only an estimated 8% of Aboriginal people in NSW report never identifying themselves as such. Evidence suggests that the highest proportion of Aboriginal people from NSW who never identify are aged 15–24 years and the propensity to identify decreases as remoteness increases [26]. Interestingly, our results demonstrate that the majority of women not recorded as Aboriginal on the birth data but reported as Aboriginal through the ERA were aged 25 and over and were from major cities or inner regional areas.

The datasets used in this study, and commonly in public health research, are not collected for research purposes and are prone to errors in data entry and linkage. There is also reason to believe that linkage errors may be more common among Aboriginal people than Non-Aboriginal people due to their names, date of birth and address information being less consistently reported across routinely collected datasets than for other Australians [1]. Consequently, all methods of ERA are susceptible to incorrectly reporting a person as Aboriginal (false positive). The ERA algorithms used in this study required a given number of records indicating Aboriginality (rather than a proportion) to assign enhanced Aboriginality, meaning the likelihood of false positives increased with the number of records included. However, this approach was taken because using the number of linked records rather than a proportion provides increased numbers of individuals being reported and this method is thought to better avoid misclassification due to an individual choosing not to be identified due to fear of discrimination [19, 27]. In order to explore the effect of increased linked records on the study population, the most restrictive algorithm only considered two datasets while the more inclusive algorithms considered five. The most restrictive algorithm was designed to minimise the introduction of bias, as most mothers in the birth data had a corresponding hospital birth record and a birth registration record, and therefore had the opportunity to be enhanced through this ERA algorithm. The algorithms used in study populations 3 and 4 included those records that were only available for women who had been admitted to an emergency department or hospital outside of the birth admission and so may have introduced some bias in the ERA, such as women with more chronic conditions (and therefore more health system contact) being included. The data and algorithms used to establish study population 2 and 4 were very similar to that outlined in the literature [4]. In this study, data relating to death was not included in any algorithm since we were interested in perinatal outcomes associated with smoking during pregnancy and inclusion of such datasets could bias the enhancement toward women who were generally more unwell.

For all of our populations, we had a constant baseline population based on the recorded Aboriginal status on the birth data. That is, no women who were recorded as Aboriginal in the birth data, were subsequently reported as non-Aboriginal in any of the enhanced populations. This assumption was made as we were looking to ‘enhance’ the known under-reporting in the birth data, and also have a constant baseline on which to build the enhanced populations.

Since mothers recorded as Aboriginal through the ERA only made up 5% and 10% of study population 2 and 3 respectively, the demographics of women from study population 1, 2 and 3 differed little from each other. A third of study population 4 consisted of mothers eligible through the ERA and consequently this influenced the demographics of this population. Of particular note was the rate of smoking during pregnancy among women from study population 4 at 44%, almost 6% lower than that in study population 1. To a lesser extent, rates of perinatal outcomes also differed depending on the study population used, results that are consistent with the literature [4]. These discrepancies reinforce the growing evidence that the approach used for reporting Aboriginality can substantially influence the rates of particular outcomes of interest [10]. However, we found that the relative risks of preterm birth, small-for-gestational age and perinatal deaths comparing smoking and non-smoking Aboriginal mothers were not influenced by the study population used. This provides new evidence to suggest that such within-group comparisons are robust to the assumptions made surrounding the reporting of Aboriginality.

The way in which indigenous peoples are recorded on health data and how these populations are quantified has important implications for reporting of health statistics around the world [28]. Enhanced reporting of indigenous peoples using data linkage is a technique used in Australia, New Zealand and Canada, all of which use slightly different approaches [4]. The findings of this study provide new evidence to suggest that within-group relative risks are robust to assumptions made surrounding the reporting of Indigenous status. However, further work comparing estimates from study populations established using different methods of enhanced reporting among this and other populations are required.

## Conclusion

It is widely acknowledged that Aboriginality is under-recorded in routinely collected datasets across Australia and enhanced reporting using linked data attempts to adjust for this. Although valuable efforts have been made, it is impossible to determine the extent to which Aboriginality is under-reported or how close any algorithm gets to the truth because there is simply no gold standard for comparison. This poses many difficulties for establishing the prevalence of health outcomes among Aboriginal people, the magnitude of the ‘gap’ between Aboriginal and non-Aboriginal Australians and tracking changes over time. Our work supports this but, more interestingly, provides evidence that estimates of within-group relative risks are reliable regardless of the assumptions made for establishing the study population through the enhanced reporting of indigenous peoples. Under-recoding of Indigenous status is not unique to Australia and these findings may have broader implications for countries that experience similar issues such as New Zealand, Canada and the United States of America.

## Statement of Ethics Approval

This study has obtained ethics approval from the Aboriginal Health & Medical Research Council of New South Wales (HREC Reference number: 1326/17).

## Appendix 1

**Figure 8: Estimated level of reporting of Aboriginal mothers on the birth data in 2010 to 2014 according to different methods of enhanced reporting of Aboriginality.**
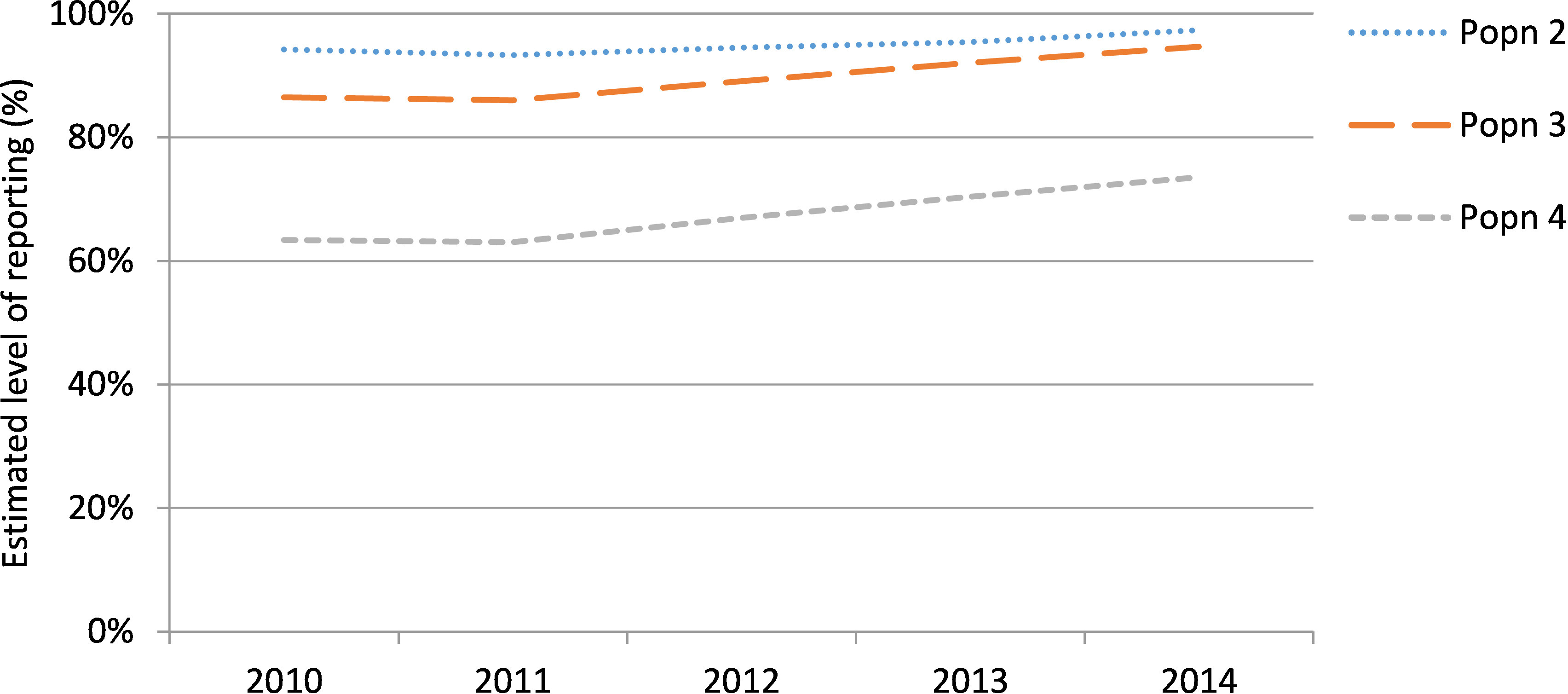
The level of reporting of Aboriginality was estimated for each year of the study period from each of the enhanced populations. This measure aims to estimate the coverage of the Aboriginal population in NSW Health data collections and represents the number of individuals recorded as Aboriginal in the baseline dataset (the birth data) as a percentage of the expected number of Aboriginal people according to the enhanced reporting process [6].
